# Testing ecological theories with sequence similarity networks: marine ciliates exhibit similar geographic dispersal patterns as multicellular organisms

**DOI:** 10.1186/s12915-015-0125-5

**Published:** 2015-02-24

**Authors:** Dominik Forster, Lucie Bittner, Slim Karkar, Micah Dunthorn, Sarah Romac, Stéphane Audic, Philippe Lopez, Thorsten Stoeck, Eric Bapteste

**Affiliations:** Department of Ecology, University of Kaiserslautern, Erwin-Schrödinger-Straße 14, Kaiserslautern, D-67633 Germany; CNRS, FR3631, Institut de Biologie Paris-Seine, Paris, F-75005 France; Sorbonne Universités, UPMC Univ Paris 06, Institut de Biologie Paris-Seine (IBPS), Paris, F-75005 France; CNRS, UMR7138, Institut de Biologie Paris-Seine, Paris, F-75005 France; CNRS, UMR 7144, Station Biologique de Roscoff, Place Georges Teissier, Roscoff, F-29680 France; Sorbonne Universités, UPMC Univ Paris 06, UMR 7144, Station Biologique de Roscoff, Place Georges Teissier, Roscoff, F-29680 France

**Keywords:** Biogeography, Microbial diversity, High-throughput sequencing, Environmental rDNA sequencing, Protist

## Abstract

**Background:**

High-throughput sequencing technologies are lifting major limitations to molecular-based ecological studies of eukaryotic microbial diversity, but analyses of the resulting millions of short sequences remain a major bottleneck for these approaches. Here, we introduce the analytical and statistical framework of sequence similarity networks, increasingly used in evolutionary studies and graph theory, into the field of ecology to analyze novel pyrosequenced V4 small subunit rDNA (SSU-rDNA) sequence data sets in the context of previous studies, including SSU-rDNA Sanger sequence data from cultured ciliates and from previous environmental diversity inventories.

**Results:**

Our broadly applicable protocol quantified the progress in the description of genetic diversity of ciliates by environmental SSU-rDNA surveys, detected a fundamental historical bias in the tendency to recover already known groups in these surveys, and revealed substantial amounts of hidden microbial diversity. Moreover, network measures demonstrated that ciliates are not globally dispersed, but are structured by habitat and geographical location at intermediate geographical scale, as observed for bacteria, plants, and animals.

**Conclusions:**

Currently available ‘universal’ primers used for local in-depth sequencing surveys provide little hope to exhaust the significantly higher ciliate (and most likely microbial) diversity than previously thought. Network analyses such as presented in this study offer a promising way to guide the design of novel primers and to further explore this vast and structured microbial diversity.

**Electronic supplementary material:**

The online version of this article (doi:10.1186/s12915-015-0125-5) contains supplementary material, which is available to authorized users.

## Background

Evaluating the patterns and processes of microbial diversity is central for understanding the ecology and evolution of life on Earth [1-3]. Currently, morphological and molecular methods have prompted a number of alternative perspectives on these issues. The extent of global dispersal versus levels of local endemism for microbial taxa [4-7], as well as the respective impacts of historical barriers to dispersal versus that of current conditions selecting among species in specific environments [8-11] were debated to explain the distribution of these organisms. These perspectives have previously been difficult to evaluate for microbial eukaryotes because extensive diversity underlies many morphospecies and most species are recalcitrant to cultivation [12,13]. Traditional morphological and molecular methods have also imposed temporal and financial limits on collecting data on total diversity within and among communities [14,15].

The recent introduction of high-throughput sequencing (HTS) methodologies provided a way to push these limits to eukaryotic microbial diversity research [16-21]. It is now possible to quickly obtain hundreds of millions of sequences. However, the development of HTS was one of those events in which a nascent technology rapidly progressed far beyond our ability to best collect and analyze the data. This situation encouraged developments to justify which DNA region should be targeted for sequencing and which primer-pairs should be used, for example, in foraminifera [22], ciliates [17,23-25], dinoflagellates [26], fungi [27], bacteria [28] and archaea [29]. There has also been little effort put into effectively exploiting the resulting millions of short HTS sequences in a statistical framework, especially when placed into the context of previous studies. Computational steps are still a major bottleneck in molecular-based environmental studies. These shortcomings have hampered the identification of novel taxa, of distribution patterns, and their biological and ecological causes.

Sequence similarity network analyses, based on sequence similarity [30-35] offer an extension to sequence clustering analyses. They should not be conflated with co-occurrence networks [36,37]. Beyond a first step of clustering, sequence similarity networks allow biologists to perform fine-grained analyses of similarities between sequences, because they exploit the information provided by the topology of weighted connections between sequences within and between clusters. Such networks rely on methods from graph theory that have recently been adapted to address an increasing number of biological questions using large molecular datasets [30,31,38-43]. With such analyses, combining massive sequence data produced from numerous studies becomes feasible, and diversity patterns can be inferred that otherwise would not be apparent from individual studies alone. For example, a count of phylotypes in two separate datasets provides no information about which of these phylotypes are the same, while sequences from different studies can be directly compared in a single network analysis.

**Figure 1:**
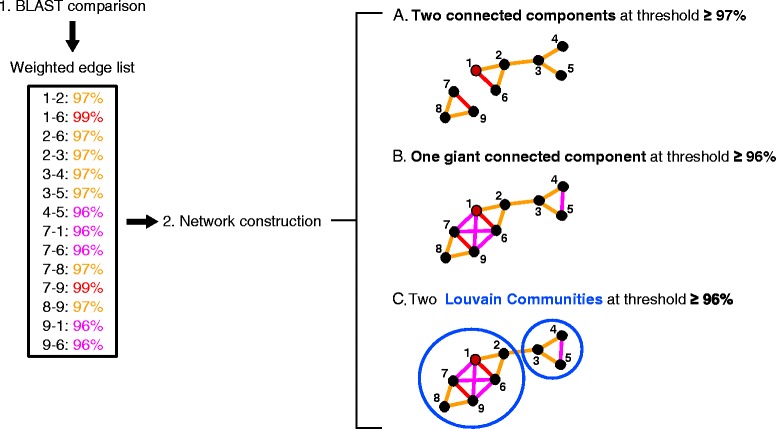
**Mock sequence similarity networks.** Weighted sequence similarity networks displaying sequences as nodes (black nodes represent environmental sequences, red nodes represent sequences of cultured ciliates), connected by edges reflecting their %ID obtained from a BLAST analysis (see list of weighted edges in which the color code reflects the %ID; red for 99%, orange for 97%, pink for 96%). The corresponding color code is used on the networks (right panel) to explore and structure the data. The mock dataset results in: **A)** two connected components when the minimum %ID required to connect two nodes is ≥97%; **B)** a single giant component when the minimum %ID required to connect two nodes is ≥96%; **C)** finer-level structure within components can be further detected using methods of community clustering, such as the Louvain Communities (see Methods). %ID, % identity.

Phylogenetic analysis of short read data has made great strides in recent years, following the development of phylogenetic placement algorithms for incorporating short read data into a reference phylogeny (for example, [44,45]). Yet, multiple alignments and tree reconstructions with hundreds of thousands to millions of environmental sequences from HTS are either slow (when accurate) or inaccurate (when fast) [44]. Furthermore, a challenge in such tools still consists in developing appropriate visualization tools and metrics for analyzing distributions of reads on computed trees [44]. The network approach offers a powerful alternative in terms of comparative and visualization strategies (Figure 1), with the benefit of introducing several informative graph-based estimates describing the relationships between sequences (Table 1), thereby offering independent ways of analyzing the distribution of microbial organisms. In other words, sequence similarity networks allow the empirical testing of aspects of the theoretical framework of microbial ecology through the exploitation of network-based properties (Table 1). Community clustering and Louvain community analyses [46] can be used to identify groups of similar sequences at various thresholds of sequence similarity. Assortativity analyses [47] can be used to define sets of sequences with distinctive characteristics. Path analyses [31] can be used to quantify divergence between sequences. All these measures offer an original path from graph theory to empirical analyses of the patterns and processes shaping microbial diversity.

**Table 1:** **Network methods and their adaptation to biological questions**

**Network term**	**Term description**	**Biological meaning in this work**
Node	Single unit of a graph	A V4 sequence
Edge	Connection between two nodes	Sequence similarity between two V4 sequences
Assortativity	Measure of the preferential connection between a set of nodes of interest	Evaluation of similarity of a set of sequences from the same habitat or location (for example, if sequences from one habitat are more similar to one another than they are similar to sequences from other habitats, their assortativity will be high. Environments with distinctive similar V4 will have such a high assortativity). See Figure 2C.
Closeness	Measure of the centrality/peripherality of a node in a network	Measure of sequence divergence relative to the rest of the dataset (for example, divergent sequences (with respect to the rest of the dataset) have a low closeness and tend to be more peripheral as they share less similarity to other sequences). See Figure 2D.
Shortest path	Shortest distance between a pair of nodes	Measure to quantify the divergence between a pair of sequences (for example, a long shortest path between an environmental sequence and a sequence from a cultured ciliate indicates a high divergence between these sequences, since these sequences are not direct neighbors in the graph). See Figure 2D.

Listed are the most important network terms introduced into microbial ecology in the framework of this study. The table indicates how these methods can be applied to HTS data to address fundamental questions on the diversity and distribution of microbial organisms. HTS, high-throughput sequencing.

In this study, we analyzed a network of 85,482 DNA and cDNA ciliate pyrosequences from the V4 region of the SSU-rDNA locus collected at eight European coastal sites from three different habitats (sediments, deep chlorophyll maximum (DCM), subsurface; for sampling details see [18,48]). We developed a protocol to: 1) detect novel diversity in our new data in the context of existing sequence data from previous environmental diversity inventories (Additional file 1, Table S1) and cultured species; and 2) test ecological theories about ciliate dispersal at multiple evolutionary levels (from conspecific to more inclusive taxonomical units). We conclude that ciliates in European coastal areas—just like bacteria, plants and animals—are under strong environmental and geographical selection at intermediate geographical scale, and agree with previous observations rejecting global dispersal hypotheses [49-53]. We argue that such a diversity structure calls for specific improved sampling strategies in future microbial community surveys.

## Results and discussion

### The large scope of network analyses

Sequence similarity networks are inclusive graphs, easily accommodating substantial molecular datasets (typically millions of sequences, but billions are possible). They provide a unified comparative framework for these sequences, which can be analyzed with the methods and tools of graph theory [54]. In such graphs, the nodes represent the objects to be compared (here ciliate V4 sequences), each pair being linked by an edge if there is significant similarity between the two corresponding nodes (here a minimum % identity (%ID), E-value, length, and alignment cover spanning over the two sequences).

**Figure 2:**
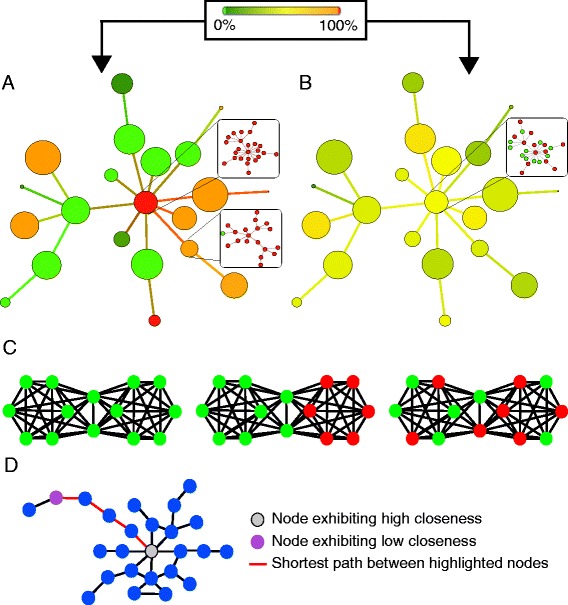
**Mock sequence similarity networks displaying endemism and cosmopolitan distribution of ciliates. A)** Schematic pattern of a GCC colored to reflect the origin of sequences in case of endemism. Each supernode corresponds to a LC consisting of a group of sequences. Exemplary LC composition is described in the insets in squared boxes. Each node color in these insets corresponds to a given hypothetical sampling depth (that is, red for **depth A**, green for **depth B**). Each supernode color represents the proportion of sequences from different sampling depths in a LC (see color bar ranging from green (100% sequences of **depth B**) to red (100% sequences of **depth A**). **B)** Schematic pattern of the same GCC colored to reflect the origin of the sequences in case of a cosmopolitan distribution of ciliates. Same color code as above. Intermediate colors of most LCs reflect the fact that highly similar sequences were detected at different depths. **C)** Schematic pattern of CCs, their node colors reflecting the origin of the sequences. As above, each color corresponds to a given sampling depth. Some structure is visible when either all similar sequences originate from the same depth (left), or when sequences from a particular depth cluster together within the component (middle). In case of a widespread dispersal across sampling depths, no structure with regard to the color code can be observed (right). **D)** Schematic pattern of a CC, describing closeness and shortest path. Two distinct nodes are highlighted. One in the center (grey) and one in the periphery (lilac) of the CC, exhibiting high and low closeness, respectively. Red edges connecting the highlighted nodes display the shortest path between these two particular nodes. See Table 1 for further explanations. CCs, connected communities; GCC, giant connected components; LC, Louvain communities.

The resulting networks provide multiple lines of evidence to analyze genetic diversity in large molecular datasets at various sequence similarity levels, hence at various taxonomical levels, and consequently to test ecological theories (Table 1). First, thresholded sequence similarity networks effectively provide a first structure of the data by partitioning it, since the continuity and discontinuity of resemblances between sequences generally produces distinct subgraphs, called connected components (Figure 1A). When numerous sequences are highly similar, as is the case for V4 sequences, connected components grow to a very large size, forming Giant Connected Components [55] (GCC, Figure 1B). Second, connected components can be further partitioned to identify densely connected regions within them, known as communities in graph theory. In each community, nodes are more connected to other nodes within the given community than to external nodes (Figure 1C). We used Louvain communities [46] (LCs) of level 1 as the finer level of sequence similarity, that is, as the tightest clusters of similar sequences. We tested whether these two methods of graph partitioning (CC and LC implementation, respectively) returned groups of sequences from similar locations, depths, or lab cultures, or whether no such geographical, habitat or ‘cultivability’ structure was observed in the network. To do this, nodes in sequence similarity networks were labelled based on sequence properties. Preferential patterns of connections can then be analyzed in these labelled graphs, using assortativity estimates that quantify to what extent sequences with the same label (for example, from a given depth or location) connect with each other rather than with differently labelled sequences [47]. For instance, geographical structuring of the data produces (1) CCs/LCs with sequences from only one sampling site (Figure 2A and C), and (2) CCs/LCs with sequences from multiple sampling sites, yet with detectable patterns of preferential connections between sequences from the same geographical location, if sequences from the same sampling site are more similar to one another than they are to sequences from other sites (Figure 2A and C).

A useful property of thresholded networks is that all sequences are not necessarily directly connected together within a CC or a LC. For instance, sequence 1 can be linked to sequence 2, itself linked to sequence 3, while sequences 1 and 3 are not directly connected because they do not share a greater similarity than the stringency threshold used to construct the network. In that particular case, the connected component (1, 2, 3) is a chain (Figure 1A). In practice, CCs (and to a lesser extent LCs) display a variety of mathematical and topological properties, ranging from chains to cliques (when all nodes are directly connected to one another), which can be exploited in comparative analyses.

Third, detailed analyses of the relationships between nodes provide additional network-based estimates of genetic diversity. In particular, the closeness of a node, quantifying its location in the graph, and the number of edges separating two given nodes can be computed [56] to compare the centrality of environmental sequences with that of sequences from cultured ciliates, and to measure their relative dissimilarity, when environmental sequences are located more than one edge apart from any sequence from cultured ciliates (that is, if sequence 1 in the chain described above was a sequence from an organism in culture and sequence 3 was a sequence from an environmental sample; these two sequences could be considered as divergent with a distance = 2 for that threshold).

In short, sequence similarity networks offer a global and inclusive framework, that allows displaying and comparing novel data with pre-existing samples and, therefore, to progress towards integrated comparative analyses of multiple data sets. Using these tools, we analyzed (1) how environmental SSU-rDNA projects have expanded our former knowledge of genetic diversity that was based on cultured ciliates, (2) how the results of different environmental SSU-rDNA surveys of microbial eukaryotic diversity compare in terms of genetic diversity, while (3) enhancing our understanding of the actual global diversity of ciliates and of its ecological structure.

### Extensive novel diversity of environmental ciliates

**Figure 3:**
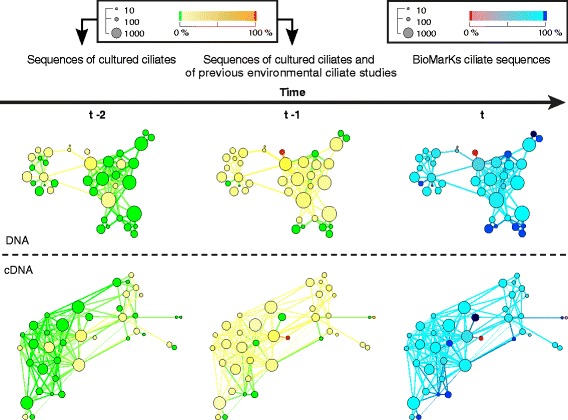
**Sequence similarity network showing the giant connected component (GCC) of each dataset.** GCC were constructed at the most inclusive sequence similarity threshold (≥85%) for DNA (above) and cDNA (below) networks. Supernodes of the GCC represent Louvain communities (LCs); the size of the supernode indicates the number of sequences in the respective LC, the color indicates the proportion of targeted sequences in the respective LC (for example, sequences from cultured ciliates at t-2, sequences from cultured ciliates and previous environmental samplings at t-1, sequences from BioMarKs at t). The figure should be read along the time of data generation axis from left to right to follow the gradual discovery of ciliates by Sanger sequencing (t-2) compared to previous environmental studies (t-1) and finally to the current 454 pyrosequencing study (t). Color of nodes at time t-2 indicates the proportion of sequences from cultured ciliates in each LC; color of nodes at time t-1 indicates the proportion of sequences from either cultured ciliates or former environmental ciliates studies in each LC; color of nodes at time t indicates the proportion of BioMarKs 454 sequences in each LC (different color code was used for reasons of visualization). GCC, giant connected components; LC, Louvain communities.

Previous environmental diversity studies [19,21,57,58] have shown that it is specifically the large proportion of low-abundance taxa in a microbial consortium, in which we find most of the novel diversity. Figure 3 shows the GCC of the DNA and of the cDNA networks constructed at the most inclusive threshold of ≥85% similarity. Densely connected regions (LCs of the GCC) of similar sequences are replaced by supernodes for display purposes. These supernodes are colored based on the percentage of sequences they comprise either from cultured organisms (t-2), from cultured and environmental sequences obtained before the BioMarKs project [48] by Sanger sequencing (t-1), or that were obtained by the BioMarKs project [48] (t) using 454 pyrosequencing. In other words, Figure 3 reveals the similarity relationships between sequences from cultured organisms and from an increasing number of environmental projects.

Ciliate sequences obtained by BioMarKs (represented by all non-pink LCs in the right graph, Figure 3 (t)) substantially increase the diversity that was known from sequences of described organisms (represented by all non-green LCs in the left graph, Figure 3 (t-2)). For cDNA sequences, the number of LCs increases 1.4 fold and for DNA 2.3 fold. When all ciliate sequences from the environmental reference database are also considered in the GCC network analyses, we find that a large proportion of BioMarKs diversity was, in fact, discovered previously in environmental molecular diversity surveys (non-pink nodes in Figure 3 (t) and non-green nodes in Figure 3 (t-1), respectively). The colors of these GCCs show that environmental projects conducted before BioMarKs mostly detected ciliates sequences that were either from the same LCs as sequences from cultured organisms, or from previously untapped LCs neighboring these communities. This observation means that rather central LCs with sequences from cultured ciliates were further enriched with environmental representatives (yellowish communities in the left graph turning more orange in the middle graph), and at the same time novel communities were discovered during these early environmental projects (green communities that were lacking any cultured member in the left graph turning yellowish in the middle graph).

**Figure 4:**
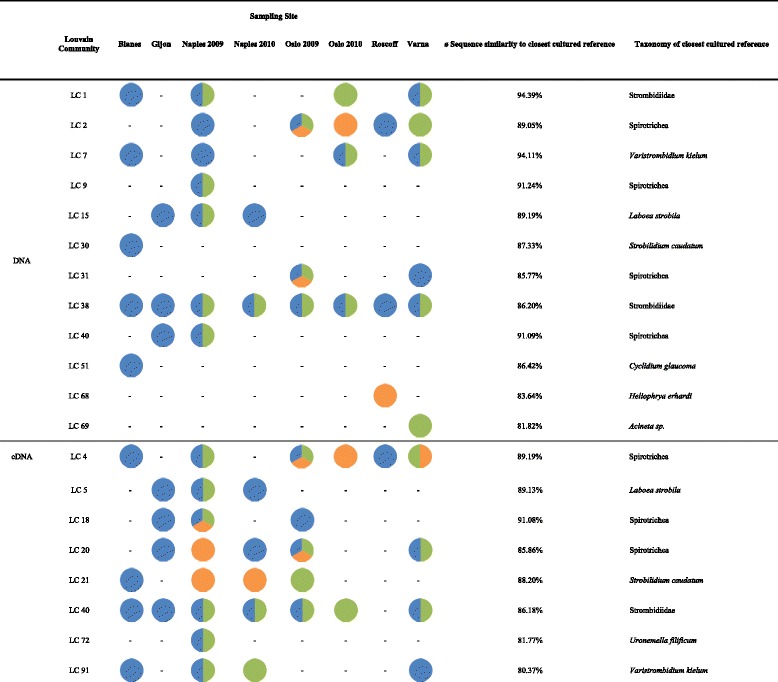
**Novel diversity identified by sequence similarity networks.** Novel diversity in the dataset was defined as every Louvain Community at the 85% similarity threshold which exclusively consisted of BioMarKs sequences and which sequences were on average less than 95% identical to any reference sequence of the cultured ciliate database. The first eight columns display the composition of the respective LC with regard to sampling sites and habitats. The colors of the circles indicate in which habitat the sequences had been detected. Blue represents subsurface, green represents DCM and orange represents sediment samples. Multicolored circles were found in more than one habitat at the same sampling site. Taxonomy is displayed to the species level of the closest cultured reference if possible. Whenever more than one closest reference was assigned to the LC, the last common taxonomic level (at least class level) is given. All V4 sequences incorporated into the listed LCs are publicly available at Figshare [59]; separate fasta files have been deposited for DNA [60] and cDNA data [61]. DCM deep chlorophyll maximum; LC, Louvain community.

The inclusion of environmental sequences from BioMarKs continued this expansion in the description of genetic diversity in ciliates, identifying more environmental representatives of previously known communities (yellowish and orange communities of the middle graph turning light blue in the right graph), and detecting novel communities (green in the middle graph, dark blue in the right graph) especially at the periphery of the graph. Figure 4 gives a more precise overview of the novel ciliate diversity revealed by the sequence similarity networks at the ≥85% threshold. Our BioMarKs data added eight distinct and previously unknown LCs to the cDNA dataset and twelve to the DNA dataset (Figure 4). The composition of these LCs does not imply the presence of a specific hotspot for the detection of novel ciliate diversity with regard to location or habitat (Figure 4). Although most of the LCs could be assigned to the class Spirotrichea, the low sequence similarities to the closest cultured references indicate that the taxonomic assignment should be taken with care.

Moreover, the structure of the sequence similarity networks also enabled us to quantify and characterize the novel diversity detected by CC analyses. At all similarity thresholds, we detected components that exclusively consisted of BioMarKs sequences (Additional file 2: Figure S1). For example, at 97% sequence similarity, a suggested approximation to discriminate ciliate species based on the V4 fragment [24], 60% of the cDNA components and 45% of the DNA components did not include any reference sequence, thus representing previously unknown ciliate diversity.

The divergence between environmental sequences and sequences from cultured ciliates was further analyzed by network path analyses (Table 1) of CC and LC. These analyses also provided evidence that environmental sequences correspond to (1) already known sequences or are highly similar to such sequences (16.6% DNA and 10.5% cDNA sequences at distance = 1, thus directly connected to a cultured ciliate sequence in the CCs at ≥97% similarity, see Additional file 3: Figure S2) but also to (2) novel sequences, expanding the known genetic diversity of ciliates (83.4% DNA and 89.5% cDNA sequences at distances >1, thus indirectly connected to cultured ciliates sequences in the CCs at ≥97% similarity, see Additional file 3: Figure S2). These novel sequences only share direct connections to other environmental sequences, and are observed even at very inclusive stringency thresholds (60% DNA sequences and 58% cDNA sequences at ≥85% similarity, see Additional file 3: Figure S2). Hence, many environmental sequences cannot be directly assigned to formerly described lineages. Results are similar for LCs (Additional file 4: Figure S3).

Likewise, the analysis of closeness values, contrasting the centrality of environmental sequences with that of sequences from cultured ciliates, provides similar conclusions. The centrality of a sequence reflects its similarity to all other sequences in the graph. The more central one sequence is, the higher its closeness and the least divergent it is with respect to all other sequences in the network. Environmental sequences, which are more divergent to sequences from organisms known from cultures, occupy the periphery of the graph. In all networks and subgraphs, sequences from cultured ciliates are more centrally located than sequences from environmental projects (Additional file 5: Table S2 and Additional file 6: Table S3). Environmental SSU-rDNA studies are thus expanding the description of genetic diversity beyond that of known sequences at all taxonomical levels, if one assumes that decreasing similarity between sequences corresponds to increasing evolutionary distance between ciliates. The only exception concerns CCs at the ≥90% similarity threshold, in which no significant differences between the closeness of sequences from cultured ciliates and from environmental studies before BioMarKs were observed. Yet, when BioMarKs sequences are considered in this particular analysis, the location of cultured ciliate sequences in central positions of CCs at ≥90% similarity is statistically supported (*P* <0.01, Kolmogorov-Smirnov test with unilateral option, see Methods; Table S2). In summary, all networks indices (paths, closeness, CCs and LCs) point to the same conclusion: network analysis unravels novel ciliate diversity, discovered by the current survey strategy.

Furthermore, our results provide an ‘historical’ perspective on the progress in diversity studies of ciliates. On one hand, they show an inherent conservative bias of environmental SSU-rDNA surveys that rediscovered ‘more of what was already known’ in terms of genetic diversity. On the other hand, they show that even after two centuries of microscopy studies and about 20 years of molecular diversity analyses, HTS sequencing approaches still reveal substantial amounts of novel diversity. Sequence similarity networks provide us with the tools to recognize the degree of this novel diversity.

Yet, how we increase our knowledge of genetic diversity of environmental ciliates clearly depends on our background knowledge: environmental diversity surveys using primers derived from cultured organisms catch in first place more of the well-known described diversity [62], rather than detecting novel peripheral groups in the networks. This suggests that the use of different sets of primers, for example, designed from alignments of the environmental sequences in these peripheral communities (Figure 4), may be an efficient opportunity to expand further the discovery of novel, divergent groups of ciliates. More precisely, groups of sequences, such as cliques [63] or peripheral sequences can be easily extracted from sequence similarity networks, producing sets of sequences with shared similarities. These sequences can be further aligned with each other to identify specific shared divergent regions, which can become new primers for upcoming analyses. This strategy could complement the extraction of SSU-rDNA genes from PCR-free shotgun metagenomic datasets, and, within these less taxonomically biased datasets, ease the selection of SSU-rDNA genes with sequence similarities to (groups of) divergent V4.

### Geographic structuring of ciliate communities

We mapped the geographic distribution of our sequences in similarity networks to test the ecological hypothesis that ciliates are cosmopolitan organisms. Assortativity analyses were used to quantify to what extent sequences from any location and depth have close homologs in more than one environmental sample of the data set. Since similar sequences are directly connected in our graphs, the larger the environmental distribution of ciliates with similar sequences, the lower the assortativity of sequences from a given location or from a given depth in the graph (since ciliates from different locations/depths with similar sequences directly connected in the graph display different labels, Figure 2B). By contrast, if ciliates with similar sequences preferentially occupy one given depth or one given location, their sequences will tend to form clusters which exclusively group sequences from that given depth or location. Sequences of ciliates with restricted geographical or habitat distribution will tend to connect with each other in our networks, and the assortativity of their respective labels will be high (Figure 2A). Hence, the proportion of connected components with significantly higher assortativity values than expected by chance for any location or depth in our graphs indicates to which extent we observed some geographical or habitat structure in the distribution of ciliates in our data set. The majority of the three tested labels for habitats, and of the eight tested labels for locations, were significantly more assortative in the analyzed networks, indicating a notable geographical and habitat structure of BioMarKs ciliate sequences (Additional file 7: Table S4). The structuring effect is especially emphasized in the cDNA dataset for networks generated at thresholds ≥95% sequence similarity (Additional file 7: Table S4).

**Figure 5:**
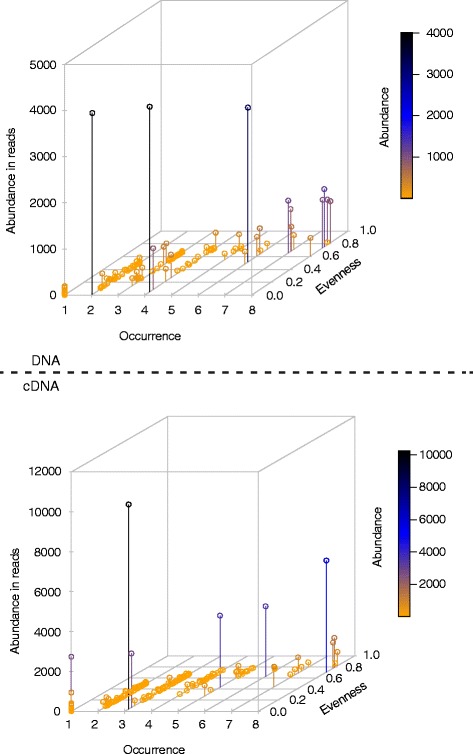
**Three-dimensional plots displaying abundance, occurrence and evenness of sequences in connected components.** Plots are based on DNA (above) and cDNA (below) networks at the most exclusive sequence similarity threshold (≥99%). Each dot (339 for DNA; 593 for cDNA) represents one CC, hence one conspecific group of ciliate sequences. Color and position of a dot on the y-axis indicates its abundance (the number of sequences in the CC). Occurrence refers to the number of sampling sites (maximum = 8) at which sequences of the CC were detected. Evenness (estimated as Simpson index) describes whether sequences of a CC are homogenously distributed across different sampling sites (SI = 0 indicates an uneven distribution, SI close to 1 indicates an even distribution). LC analyses revealed similar results (see Additional file 8: Figure S4). CC, connected components; LC, Louvain communities.

Furthermore, the global dispersal hypothesis of individual taxa was not supported by our data. On the contrary, only a maximum of 2% of all CCs at the ≥99% similarity threshold include sequences from all sampling sites (Figure 5). Figure 5 shows the abundance of sequences for each CC (results for LC were similar, see Additional file 8: Figure S4), the number of locations in which sequences from these clusters were found (occurrence), and whether these sequences were evenly distributed across the locations in which they were detected, or rather dominant at some specific location (evenness). The vast majority of all components (between 92% and 95%) is restricted to four or fewer sampling sites and is found at low abundance. Only a few groups of ciliates were found in all samples at high abundance with an even distribution of sequences. This further fuels the moderate endemicity hypothesis for protists [7], which was also supported in previous morphospecies-based studies [52,53,64,65] as well as in environmental gene-based diversity inventories [49-51]. Oxygen and salinity were identified in these and other studies as major dispersal barriers for protists (for example, [66]) and also for bacteria [67]. Indeed, oxygen-depletion may be a major factor that distinguishes the water samples from the sediment samples in our BioMarKs data set (most sediments generally become anoxic within a few millimeters below the surface [68]). Likewise, salinity differences were noteworthy among the BioMarKs sampling sites, ranging from 16.73 psu (practical salinity units) in the Black Sea to 37.93 psu in the Mediterranean Sea [48]. Additional possibilities are discussed elsewhere as potential dispersal barriers for microbes; for example, constraints in active and passive dispersal [11,69], and also the success rate of colonization (establishment of a stable population) in the new environment [70]. The latter is influenced by a wealth of biotic and abiotic processes [71]. Although there may be limits to the biogeography of many protists, ciliates, like bacteria [72], may have a common core of diversity present at very low levels, a phenomenon that could be tested with HTS for example. Finally, while geographically widely dispersed components include slightly more abundant sequences, not all abundant sequences are widely dispersed. For instance, the component with the highest abundance in Figure 5 (n = 10,255) includes sequences from only three different sampling sites.

### Habitat selection on ciliate populations

**Figure 6:**
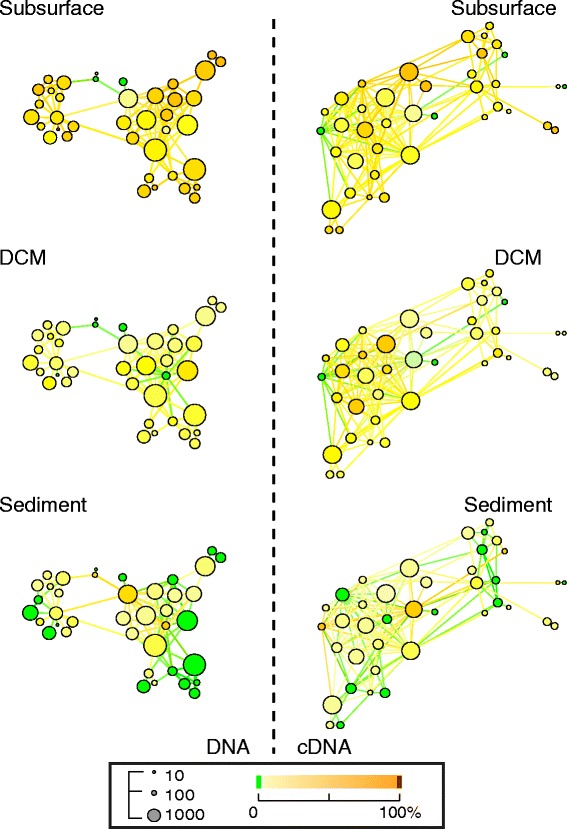
**Sequence similarity network showing the proportion of sequences from each habitat in the GCC of DNA and cDNA datasets.** GCCs, decomposed in LCs as in Figure 3 are shown for DNA (left) and for cDNA (right) networks. Network graphs are organized from top to bottom to compare the proportion of sequences from subsurface and DCM habitats with those from sediment habitats. The color of nodes (green meaning 0%, red meaning 100%) indicates the proportion of sequences from subsurface habitats in each LC (top); the proportion of sequences from DCM habitats in each LC (middle); and the proportion of sequences from sediment habitats in each LC (bottom). DCM, deep chlorophyll maximum; GCC, giant connected components; LC, Louvain communities.

Besides network components that are geographically restricted to one or a few specific sampling sites (Figure 5), the effect of environmental selection is also shaping ciliate community composition (Additional file 7: Table S4, discussed above). The GCC of the DNA and cDNA networks at ≥85% similarity clearly illustrate this situation, in spite of their low stringency threshold (Figure 6), ciliates in sediments exhibit sequences that are different from ciliates detected in the water column (subsurface and DCM). While subsurface and DCM sequences are often found in the same clusters, only a few of these clusters also include sediment sequences. In Figure 6 this observation is highlighted by the large number of LCs which are non-green in both the top (subsurface) and middle (DCM) graphs, compared to the increasing number of green LCs in the bottom (sediment) graphs.

This result of contrasting sequences from sediments and water column also occurs for conspecific ciliates (defined here as groups of ciliates with ≥97% similar sequences). Using a sequence similarity threshold of 97% for CC analyses, we found that 50% of all sediment cDNA components are exclusive to this habitat, while only 4% and 9% are exclusive to the subsurface and the DCM (Additional file 9: Figure S5). A similar trend was observed in DNA components, of which 30% were sediment-exclusive, 12% subsurface-exclusive and 7% DCM-exclusive (Additional file 9: Figure S5). Habitat specialization of marine protistan species at an even closer geographical proximity was also reported by Orsi and colleagues [50], who could relate their findings to a distinct geochemical gradient. In comparison, our results show a less pronounced level of habitat specialization along the water column, which we explain by the lack of a geochemical gradient and, thus, the less exigency for organisms to adapt. On the other hand, the high level of ciliate organisms which are specialized to sediment habitats is not unexpected, because a psammophilic life style requires different adaptations than a planktonic life style [73]. In a morphology-based approach combining publicly available ciliate data of 17 globally distributed marine benthic sampling sites, Azovsky and Mazei [74] could show that one quarter of the observed ciliate species were only detected at one single sample site and every second species occurred at not more than three different sites. Likewise, an environmental SSU-rDNA study [75] revealed that geographical structures at the sea-floor can act as biogeographical barriers and lead to distinctive benthic microbial communities. A more fragmented distribution of locally restricted benthic ciliate species, induced by geographical structures, such as the Strait of Gibraltar, could explain the comparably high level of exclusive CCs from the sediment observed in the current network approach.

## Conclusions

### Network analysis in microbial biogeography

We present network analyses as highly efficient tools to exploit massive HTS datasets and identify novel diversity. Further, network analyses appear as a useful means to address fundamental subjects in ecology using environmental diversity inventories. Such subjects include the analyses of microbial distribution patterns that allow us to draw conclusions about the underlying biological causes. For instance, while closely related ciliate sequences grouped together in our networks, the in-depth analyses of these groups showed that they are structured with regard to habitat or geographic location. With a broad ecological perspective, this can be interpreted as taxonomically closely related groups (depending on the resolution of sequence similarity we choose) which form distinct subcommunities in different habitats or at different geographic locations in European coastal waters. At the same time, we also find highly adapted groups which are restricted to one specific habitat or one location. This is similar to patterns observed in animals and plants [76] and confirms observations on ciliate dispersal patterns from other oceanographic areas [49,50]. Using network analyses of molecular markers, important theories can thus be tested: the technology for massive data production at an affordable price is in place, and powerful inclusive methods are available.

As we integrated data from multiple samples into the same comparative context, we must also conclude that surveying ciliate diversity appears more as a challenge than ever. The restriction of most taxa to one or a few different geographic locations and habitats, supported by all network estimates and for both DNA and cDNA sequences, means that we may have to bury our hopes to exhaust the ciliate (and most likely the microbial) diversity of an environment using available ‘universal’ primers and local in-depth sequencing [62]. Network analyses of sequence data can still contribute to explore such a structured vast microbial diversity. Sequence similarity networks provide all the tools needed to detect exclusively environmental clusters (CC or LC) and/or groups of sequences which are distant from previously described diversity. Alignment of groups of novel, peripheral, environmental sequences thus revealed by network analyses can guide the design of new sets of primers which will be useful to expand our knowledge of microbial diversity, moving it further away from the current *status quo* and its logical starting point: the sequences of cultured organisms.

The creation of distinct primers should compensate for the inherent tendency of recent environmental rDNA surveys whose findings gravitate around the same groups of organisms [62]. We encourage readers who want more than ‘more of the same’ from diversity surveys, and to enhance the scale at which ecological theories about microbial diversity can be tested, to experience this kind of inclusive network-based strategy.

## Methods

### Constitution of the dataset

A first reference database of ciliate sequences was built including 308 V4 SSU-rDNA sequences from GenBank. Only sequences from cultured and morphologically identified isolates were selected. Additionally a second reference database of 82,560 environmental ciliate SSU-rDNA sequences from earlier diversity studies was generated from GenBank. Moreover, environmental samples were collected at eight European marine coastal sites as part of the biodiversity of marine eukaryotes project (BioMarKs [48]). Information about sampling procedure, filter preparation, extraction of nucleic acids, sequencing strategy, sequence quality check and taxonomic assignment (on the phylum level) of the samples can be found in Logares *et al.* (2014) [18]. Stringent cleaning options were used (exact primer matching, removing low quality score sequences using sliding windows of 50 bp, and chimera checking) to guarantee the quality and reliability of the final dataset. Each sequence was labelled based on its origin (cultured organism, earlier environmental project, BioMarKs environmental sample), depth in the water column (subsurface, DCM, sediment), and sampling site of the BioMarKs project [48]. In all environmental datasets, only sequences which could be assigned to a culture sequence belonging to the phylum Ciliophora and had at least 300 bp in length were considered, amounting to 85,482 BioMarKs ciliate V4 SSU-rDNA sequences. Furthermore, datasets were dereplicated (when identical, only unique sequences were kept for each sampling site in the BioMarKs database), which resulted in a final dataset of 25,842 unique BioMarKs sequences (8,931 DNA and 16,911 cDNA sequences, respectively), 308 ciliate culture sequences and 928 ciliate sequences of earlier environmental studies (listed in Additional file 1: Table S1). Sequence data used in this analysis are publicly available at Figshare [59,77]. Additionally, we deposited one table listing all BioMarKs ciliate sequences and their respective properties [78] and one table listing all cultured ciliate reference sequences [78]. Two fasta files that include all novel ciliate diversity sequences as listed in Figure 4 are also available and may be used to design novel primers (DNA fasta file: [60]; cDNA fasta file: [61]). For better understanding of the deposited data, we also provide a manual on how to navigate in our files [79].

### Graph construction

Two types of sequence similarity networks (Figure 1) were constructed in this study to compare the consilience between the information within BioMarKs DNA sequences on the one hand and RNA sequences on the other hand. We used (1) a network with BioMarKs DNA plus reference sequences (10,167 sequences) and (2) a network with BioMarKs cDNA plus reference sequences (18,147 sequences). In both cases, sequences were used as an input file for EGN [80] choosing the following options: BLASTn E-value <1e-5; minimum hit identity threshold 60%; minimum hit length 40% of the smallest homolog. This protocol excluded singletons and resulted in disconnected networks at various sequence similarity thresholds (85%, 90%, 95%, 96%, 97%, 98%, 99%). Meaning that in a network with a sequence similarity threshold of 97%, two nodes (where each node represents one sequence from the dereplicated DNA or cDNA dataset, respectively) are connected by an edge only if they share a sequence similarity of at least 97% (Figure 1A). As a consequence, sequence similarity networks provide a first clustering, producing connected components (CCs), that is, sets of connected sequences isolated from the rest of the graph (Figure 1A). A second level of sequence clustering was achieved using the Louvain method [46] (default parameters, first level, for example, the most fine-grained resolution) (Figure 1C). This method identifies densely connected nodes in a graph, and aggregates these nodes into clusters (Louvain communities (LCs)).

The EGN output which served as the basis for all subsequent network analyses in the context of our work is publicly available at Figshare [81].

### Graph display

The largest connected component of the sequence similarity networks was found at ≥85% similarity and its LCs were displayed using Gephi [82] (Yifan Hu multilevel layout). Each LC is represented by a supernode. Two LCs are connected to another, when there is at least one connection between the two sets of sequences belonging to that pair of LC (Figures 3 and 6). The number of sequences in a LC is represented by the supernode size. LCs were colored based on their proportion of sequences with a label of interest (for example, cultured or environmental sequence, habitat affiliation) to display the structure of genetic diversity of ciliates (Figures 3 and 6).

### Graph analyses

Clusters exclusively comprising sequences with the same label (for example, BioMarKs sequences or cultured ciliate sequences) were quantified. Using Igraph library scripts [83] in the R statistical computing environment [84], we applied two measures of dissimilarity between BioMarKs sequences, environmental sequences obtained in previous projects, and sequences from cultured ciliates. First, we used the minimal shortest path between all pairs of nodes of interest, expressed in number of edges (Table 1, Figure 2D); in other words, the minimal number of edges that must be crossed to connect any environmental node (that is, a BioMarKs sequence or a sequence from a previous environmental project) and its closest node of a cultured ciliate sequence (infinite when no such path existed). Second, we used the closeness of environmental nodes and nodes from cultured ciliates in our graphs (Table 1, Figure 2D). This closeness quantifies the centrality of sequences in the graph: more peripheral sequences are more different from most other sequences of the dataset. The distributions of closeness values for these groups of nodes were compared using the Kolmogorov-Smirnov test (*P* <0.05 and *P* <0.01) with the unilateral option (test 1: cultured sequences *versus* sequences from former environmental studies; test 2: cultured sequences *versus* all environmental sequences (former environmental studies plus BioMarKs)).

### Testing the global dispersal theory

Two distinct measures were used to test whether all groups of ciliates with similar V4 DNA or cDNA sequences were found at all sampling sites. First, assortativity—a measure for the tendency of nodes with the same label (or of nodes without that label) to preferentially connect with one another in the graph—was computed for each label for each CC and LC. Assortativity was defined as in Newman (2003) [47], for two categories (nodes of the targeted label—for example, a given habitat—and other nodes). An assortativity coefficient r = 0 means that edges between the two categories are distributed randomly between the two categories. A positive coefficient indicates that nodes of the same category (that is, same depth or location) tend to be linked together, while a negative one indicates that nodes of different categories tend to be linked together. Therefore, under total endemism, patterns as in Figure 2A are expected in a graph. Sequences from a given depth or location cluster preferentially together and fall within the same exclusive region of the graph. By contrast, in the case of a cosmopolitan distribution of ciliates, similar sequences can be found in all depths and locations, producing a pattern such as that of Figure 2B.

Statistical significance of the assortativity values was assessed by randomly shuffling the labels over all sequences for each CC and LC while keeping the same network topology. Assortativity was computed for each label before and after the shuffling. Distributions of original and randomized assortativity values were compared with a Kolmogorov-Smirnov test (*P* <0.05) to test whether observed assortativity values were significantly greater than by chance.

Second, for each cluster of sequences in our ≥99% similarity networks, likely grouping sequences of the same ciliate species, we plotted the abundance of that cluster (measured as the number of sequences), the occurrence of that cluster (measured as the number of sampling sites at which these sequences were detected), and the evenness of that cluster, measured by the Simpson index (SI) $\left[SI=1-{\displaystyle \sum_{i=1}^{objects}{p}_i^2}\right]$ in which *p*_i_ corresponds to the proportional abundance of units (here: sequences) counted in object *i* (here: each sampling site) [85]. The value of SI ranges between 0 and an upper limit of 1-1/N (N being the total number of objects). A cluster of sequences which is perfectly homogeneously distributed among the sampling sites will reach a SI close to 1.

## Additional files

Additional file 1: Table S1. Publicly available SSU-rDNA sequence data included into the environmental reference database. The table shows which studies had been included into the environmental reference database of our sequence similarity network approach. The first column gives the number of V4-SSU-rDNA sequences of each study which fulfilled our requirements and were thus incorporated into the environmental reference database. Additional file 2: Figure S1. Proportion of CCs (above) and LCs (below) in DNA and cDNA networks which exclusively contained BioMarKs sequences. DNA data are shown in red, cDNA data in blue. Composition of each CC and LC was analyzed at all sequence similarity thresholds to detect references from cultured ciliate sequences and sequences of former environmental studies. Proportion of CCs and LCs displayed here only contain sequences of BioMarKs project. The more inclusive the threshold (that is, the lower the sequence similarity), the fewer CCs and LCs can be detected. Thus, the proportion of CCs and LCs exclusively containing BioMarKs sequences decreases with lower similarity thresholds. In comparison to DNA networks, a higher proportion of exclusive CCs and LCs can be found in the cDNA networks, except for the most inclusive similarity threshold (≥85%). Additional file 3: Figure S2. Network path analyses of CCs. For each environmental node at all sequence similarity thresholds, the shortest path to a reference node (node of a cultured ciliate) has been calculated. Shortest path analyses describe the minimum number of edges linking the initial node with the target node. Distance of ‘1’ to a reference node means that the environmental node is directly connected to the reference node (cultured ciliate). Distance of ‘inf’ (means infinite) to a reference node means that the environmental node is in a CC without any reference node (cultured ciliate), hence no shortest path can be calculated. The proportion of environmental nodes for which a path length of ‘inf’ is reported, decreases with the sequence similarity threshold. Numbers on top of each plot indicate the abundance of CCs regarding the sequence similarity threshold. Additional file 4: Figure S3. Network path analyses of LCs. For each environmental node at all sequence similarity thresholds, the shortest path to a reference node (node of a cultured ciliate sequence) has been calculated. Shortest path analyses of LCs revealed similar patterns as the shortest path analyses of CCs (Additional file 3: Figure S2). Additional file 5: Table S2. Kolmogorov-Smirnov (KS) tests comparing closeness distributions in DNA networks. At all sequence similarity thresholds and for both CCs and LCs two independent KS-tests were performed: 1) to test if the closeness of nodes of cultured ciliates (C_cultured_) was higher than the closeness of nodes from former environmental studies before 454 sequencing (C_former Env_) and 2) to test if the closeness of C_cultured_ was higher than the closeness of all environmental nodes (C_Env_: nodes from former environmental studies plus BioMarKs nodes). Closeness can be understood as a measurement of centrality in a network (that is, the higher the closeness, the more central the nodes will be located). For all but one case it could be confirmed that C_cultured_ was significantly higher than C_former Env_ (*P* <0.05 or *P* <0.01). Compared to C_Env_, C_cultured_ was always significantly higher. Additional file 6: Table S3. KS-tests comparing closeness distributions in cDNA networks. Results and patterns of the KS-tests on closeness in DNA networks (Additional file 5: Table S2) are confirmed. Additional file 7: Table S4. KS-test comparing the distribution of assortativity in gene similarity networks. Assortativity describes the tendency of nodes to be connected to nodes of the same label. Two independent groups of labels (three habitats, eight locations) were tested for DNA and cDNA networks at each sequence similarity threshold. Using a one-sided KS-test we analyzed if the distribution of assortativity values of each respective label was significantly greater (*P* <0.05) than expected by chance. Thus the nodes of the respective label were more likely to connect with each other than to connect to nodes of another label. Additional file 8: Figure S4. Three-dimensional plots displaying abundance, occurrence and evenness of sequences in LCs. Plots are based on DNA (above) and cDNA (below) networks at the most exclusive sequence similarity threshold (≥99%). Each dot (370 for DNA; 670 for cDNA) represents one LC. Additional file 9: Figure S5. Composition of CC in DNA (above) and cDNA (below) gene similarity networks. Each CC at each similarity threshold was analyzed to highlight the proportion of nodes, which derived from the three investigated habitats in this study (Subsurface, DCM, Sediment). The graphs show the proportion of all CCs, which exclusively consist of nodes of the same habitat. In all networks the highest proportion of exclusive CCs was found for sediment habitats (orange), in some networks providing more than 50% of all CCs. The proportion of DCM (green) and subsurface (blue) CCs never reached more than 15.4% and 16.4%, respectively, of the total number of CCs in the respective network. In the DNA networks an increase of CCs harboring exclusively nodes of DCM habitats could be observed with decreasing sequence similarity. The reason for this is a few exclusive DCM CCs which do not merge with any other CC although the threshold gets more inclusive and fewer CCs in total can be found. Consequently—as the reported values are proportions, not absolute numbers—more CCs can be detected in proportion to the total number of CCs.
