# Supplementary material for: Testing ecological theories with sequence similarity networks: marine ciliates exhibit similar geographic dispersal patterns as multicellular organisms
Source: BMC Biol. 2015 Feb 24;13:16. doi: 10.1186/s12915-015-0125-5 (PMC4381497; doi:10.1186/s12915-015-0125-5)
Supplement: Additional file 1: Table S1. — Publicly available SSU-rDNA sequence data included into the environmental reference database. The table shows which studies had been included into the environmental reference database of our sequence similarity network approach. The first column gives the number of V4-SSU-rDNA sequences of each study which fulfilled our requirements and were thus incorporated into the environmental reference database. [file 12915_2015_125_MOESM1_ESM.docx]

| **Sequences in Environmental Reference Database** | **Study title** | **Author** | **Publication** |
| --- | --- | --- | --- |
| 1 | Analysis of the eukaryotic community and metabolites found in clay wall material used in the construction of traditional Japanese buildings | Kitajima *et al.* | *Bioscience Biotech Biochem* 2010, **74**(10):2083-2086 |
| 40 | Bait worm packaging as a potential vector of invasive species | Haska *et al.* | *Biol Invasions* 2012, **14**(2): 481-493 |
| 13 | Chrysophytes and other protists in High Arctic lakes: molecular gene surveys, pigment signatures and microscopy | Charvet *et al.* | *Polar Biol* 2012, **35**(5):733-748 |
| 37 | Comparative analysis between protist communities from the deep-sea pelagic ecosystem and specific deep hydrothermal habitats | Sauvadet *et al.* | *Environ Microbiol*  2010, **12**(11):2946-2964 |
| 32 | Comparison of wintertime eukaryotic community from sea ice and open water in the Baltic Sea, based on sequencing of the 18S rRNA gene | Majaneva *et al.* | *Polar Biol* 2012, **35**(6):875-889 |
| 2 | Compositional differences in particle-associated and free-living microbial assemblages from an extreme deep-ocean environment | Eloe *et al.* | *Environ Microbiol Rep* 2011,**3**(4):449-458 |
| 3 | Diversity and community dynamics of protistan microplankton in Sagami Bay in spring season revealed by 18S rRNA gene clone analysis | Kok *et al.* | *Plank Benth Res* 2012, **7**(2):75-86 |
| 5 | Diversity and vertical distribution of microbial eukaryotes in the snow, sea ice and seawater near the north pole at the end of the polar night | Bachy *et al.* | *Front Microbiol* 2011, **2**(106):doi:10.3389/fmicb.2011.00106. |
| 25 | Diversity estimates of microeukaryotes below the chemocline of the anoxic Mariager Fjord, Denmark | Zuendorf *et al.* | *FEMS Microbiol Ecol* 2006, **58**(3):476-491 |
| 3 | Diversity of microbial eukaryotes in Kongsfjorden, Svalbard | Luo *et al.* | *Hydrobiol* 2009, **636**(1):233-248 |
| 83 | Effect of Oxygen Minimum Zone Formation on Communities of Marine Protists | Orsi *et al.* | *ISME* 2012, **6**(8):1586-1601 |
| 13 | Eukaryotic phylotypes in aquatic moss pillars inhabiting a freshwater lake in East Antarctica, based on 18S rRNA gene analysis | Nakai *et al.* | *Polar Biol* 2012, **35**(10):1495-1504 |
| 5 | Genetic diversity of small eukaryotes in lakes differing by their trophic status | Lefranc *et al.* | *Appl Environ Microbiol* 2005, **71**(10):5935-5942 |
| 11 | Genetically diverse and highly selective diet of the copepod *Acartia tonsa* in the natural environment | Lin *et al.* | Direct Submission 2011 |
| 1 | Large-scale patterns in biodiversity of microbial eukaryotes from the abyssal sea floor | Scheckenbach *et al.* | *Proc Natl Acad Sci USA* 2010, **107**(1):115-120 |
| 9 | Microbial community structure of a slow sand filter schmutzdecke: a phylogenetic snapshot based on rRNA sequence analysis | Wakelin *et al.* | *Water Sci Technol Water Supply* 2011, **11**(4):426-436 |
| 44 | Microbial diversity analysis using pyrosequencing of small-subunit ribosomal RNA without PCR amplification | Quan ZX | Direct Submission 2010 |
| 22 | Microbial eukaryotes in the hypersaline anoxic L'Atalante deep-sea basin | Alexander *et al.* | *Environ Microbiol* 2009, **11**(2):360-381 |
| 26 | Microeukaryote community patterns along an O2/H2S gradient in a supersulfidic anoxic fjord (Framvaren, Norway) | Behnke *et al.* | *Appl Environ Microbiol* 2006, **72**(5):3626-3636 |
| 14 | Microeukaryotic diversity in marine environments, an analysis of surface layer sediments from the East Sea | Park *et al.* | *J Microbiol* 2008, **46**(3):244-249 |
| 31 | Microfaunal indicators, Ciliophora phylogeny and protozoan population shifts in an intermittently aerated and fed bioreactor | Ntougias *et al.* | *J Hazard Mater* 2011, **186**(2-3):1862-1869 |
| 49 | Molecular characterization of ciliate diversity in stream biofilms | Dopheide *et al.* | *Appl Environ Microbiol* 2008, **74**(6):1740-1747 |
| 88 | Molecular characterization of ciliate diversity within constructed wetlands revealed unexpected high genetic variation in rDNA sequences | Haentzsch *et al.* | Direct Submission 2010 |
| 7 | Molecular diversity of the syndinean genus *Euduboscquella* based on single-cell PCR analysis | Bachvaroff *et al.* | *Appl Environ Microbiol* 2012, **78**(2):334-345 |
| 46 | Molecular evidence that phylogenetically diverged ciliates are active in microbial mats of deep-sea cold-seep sediment | Takishita *et al.* | *J Eukaryot Microbiol* 2010, **57**(1):76-86 |
| 1 | Novel eukaryotes from the permanently anoxic Cariaco Basin (Caribbean Sea) | Stoeck *et al.* | *Appl Environ Microbiol* 2003, **69**(9):5656-5664 |
| 11 | Novel eukaryotic lineages inferred from small-subunit rRNA analyses of oxygen-depleted marine environments | Stoeck *et al.* | *Appl Environ Microbiol* 2003, **69**(5):2657-2663 |
| 9 | Novel kingdom-level eukaryotic diversity in anoxic environments | Dawson *et al.* | *Proc Natl Acad Sci USA* 2002, **99**(12):8324-8329 |
| 4 | Phylogenetic diversity of 18S rRNA from uncultured small eukaryotes in Lake Kusaki | Fujimoto N | Direct Submission 2011 |
| 11 | Phylogenetic diversity of nanoplankton in Sargasso Sea Eddies | Armbrust *et al.* | Direct Submission 2006 |
| 7 | Protist community composition during spring in an Arctic flaw lead polynya | Terrado *et al.* | *Polar Biol* 2011, **34**(12):1901-1914 |
| 15 | Protist diversity in suboxic and sulfidic waters of the Black Sea | Wylezich *et al.* | *Environ Microb* 2011, **13**(11):2939-2956 |
| 16 | Protistan Community Patterns Within the Brine and Halocline of Deep Hypersaline Anoxic Basins | Edgcomb *et al.* | *Extremophiles* 2009, **13**(1):151-167 |
| 87 | Protistan microbial observatory in the Cariaco Basin, Caribbean. I. Pyrosequencing vs Sanger insights into species richness | Edgcomb *et al.* | *ISME* 2011, **5**(8):1344-1356 |
| 7 | Small planktonic communities in a meso-eutrophic lake (Esch-sur-Sure, Luxembourg) investigated by flow cytometry and cloning-sequencing approaches | Masquelin *et al.* | Direct Submission 2010 |
| 10 | Spatial and temporal dynamics of the microbial community in the Hanford unconfined aquifer | Lin *et al.* | *ISME* 2012, **6**(9):1665-1676 |
| 98 | Spatio-temporal variations in protistan communities along an O/HS gradient in the anoxic Framvaren Fjord (Norway) | Behnke *et al.* | *FEMS Microbiol Ecol* 2010, **72**(1):89-102 |
| 1 | Structured multiple endosymbiosis of bacteria and archaea in a ciliate from marine sulfidic sediments: A survival strategy in low oxygen, sulfidic sediments? | Edgcomb *et al.* | *Front Microbiol* 2011, **2**(55):doi:10.3389/fmicb.2011.00055. |
| 3 | Study of genetic diversity of eukaryotic picoplankton in different oceanic regions by small-subunit rRNA gene cloning and sequencing | Díez *et al.* | *Appl Environ Microbiol* 2001, **67**(7):2932-2941 |
| 6 | The molecular diversity of freshwater picoeukaryotes from an oligotrophic lake reveals diverse, distinctive and globally dispersed lineages | Richards *et al.* | *Environ Microbiol* 2005, **7**(9):1413-1425 |
| 6 | The response of marine picoplankton to ocean acidification | Newbold *et al.* | *Environ Microbiol* 2012, **14**(9):2293-2307 |
| 1 | Unexpected importance of potential parasites in the composition of the freshwater small-eukaryote community | Lepère *et al.* | *Appl Environ Microbiol* 2008, **74**(10):2940-2949 |
| 16 | Use of flow cytometric sorting to better assess the diversity of small photosynthetic eukaryotes in the English Channel | Marie *et al.* | *FEMS Microbiol Ecol* 2010, **72**(2):165-178 |
| 9 | Use of stable isotope-labelled cells to identify active grazers of picocyanobacteria in ocean surface waters | Frias-Lopez *et al.* | *Environ Microbiol* 2009, **11**(2):512-525 |
