# Supplementary figures and images for: Testing ecological theories with sequence similarity networks: marine ciliates exhibit similar geographic dispersal patterns as multicellular organisms
Source: BMC Biol. 2015 Feb 24;13:16. doi: 10.1186/s12915-015-0125-5 (PMC4381497; doi:10.1186/s12915-015-0125-5)

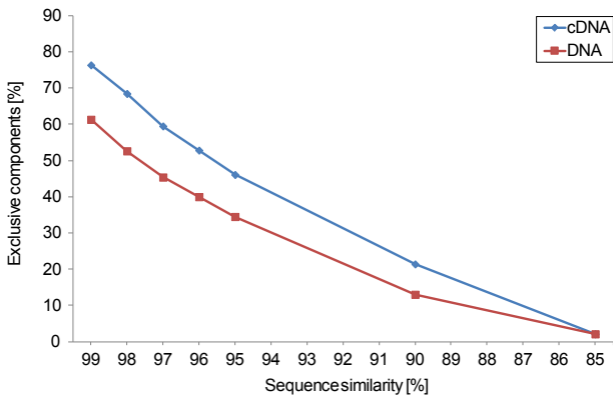

CC

LC

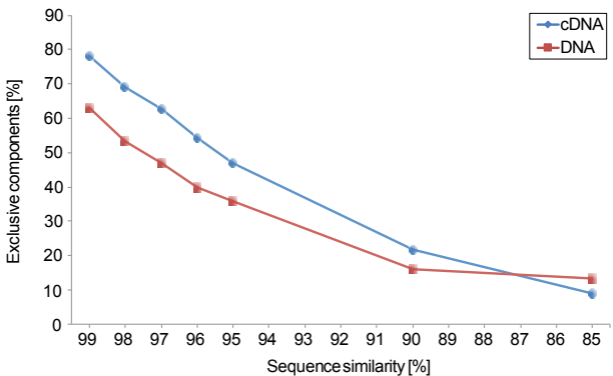

Supplement: Additional file 2: Figure S1. — Proportion of CCs (above) and LCs (below) in DNA and cDNA networks which exclusively contained BioMarKs sequences. DNA data are shown in red, cDNA data in blue. Composition of each CC and LC was analyzed at all sequence similarity thresholds to detect references from cultured ciliate sequences and sequences of former environmental studies. Proportion of CCs and LCs displayed here only contain sequences of BioMarKs project. The more inclusive the threshold (that is, the lower the sequence similarity), the fewer CCs and LCs can be detected. Thus, the proportion of CCs and LCs exclusively containing BioMarKs sequences decreases with lower similarity thresholds. In comparison to DNA networks, a higher proportion of exclusive CCs and LCs can be found in the cDNA networks, except for the most inclusive similarity threshold (≥85%). [file 12915_2015_125_MOESM2_ESM.pdf]

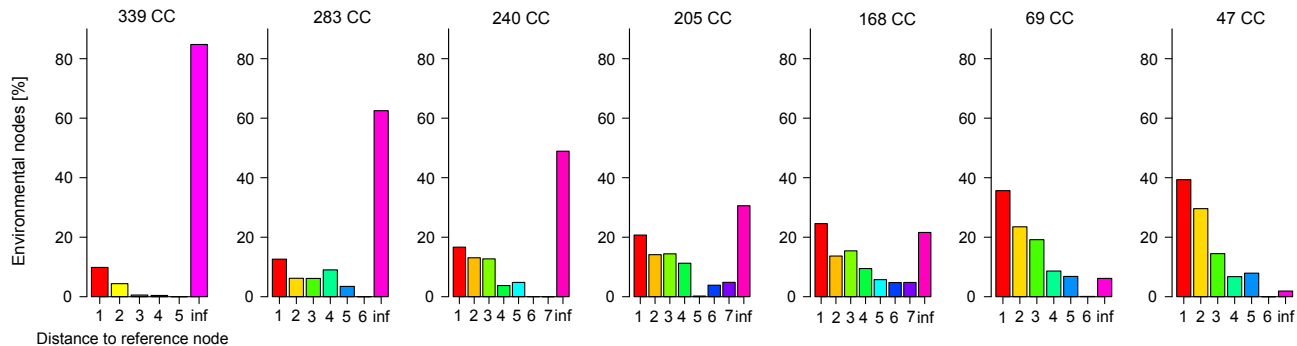

## DNA

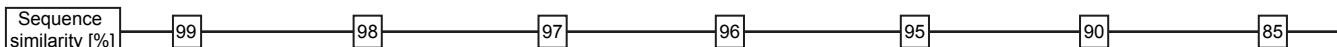

## cDNA

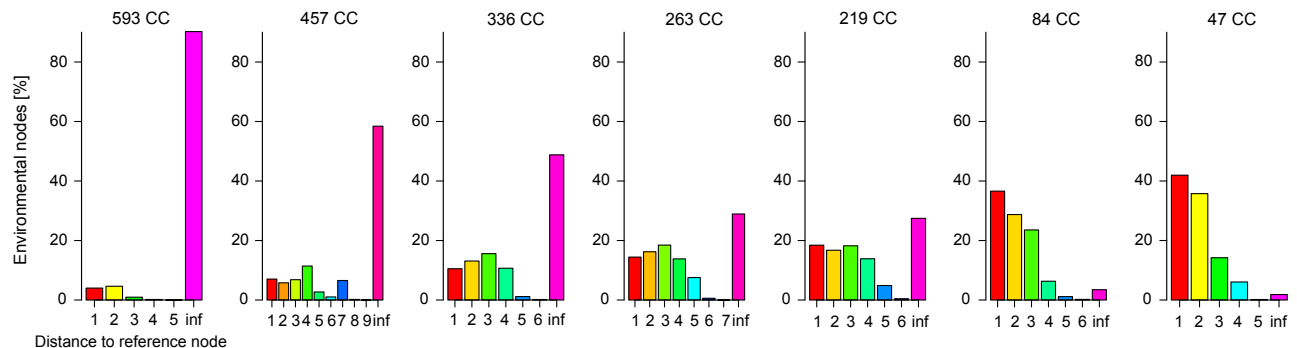

Supplement: Additional file 3: Figure S2. — Network path analyses of CCs. For each environmental node at all sequence similarity thresholds, the shortest path to a reference node (node of a cultured ciliate) has been calculated. Shortest path analyses describe the minimum number of edges linking the initial node with the target node. Distance of ‘1’ to a reference node means that the environmental node is directly connected to the reference node (cultured ciliate). Distance of ‘inf’ (means infinite) to a reference node means that the environmental node is in a CC without any reference node (cultured ciliate), hence no shortest path can be calculated. The proportion of environmental nodes for which a path length of ‘inf’ is reported, decreases with the sequence similarity threshold. Numbers on top of each plot indicate the abundance of CCs regarding the sequence similarity threshold. [file 12915_2015_125_MOESM3_ESM.pdf]

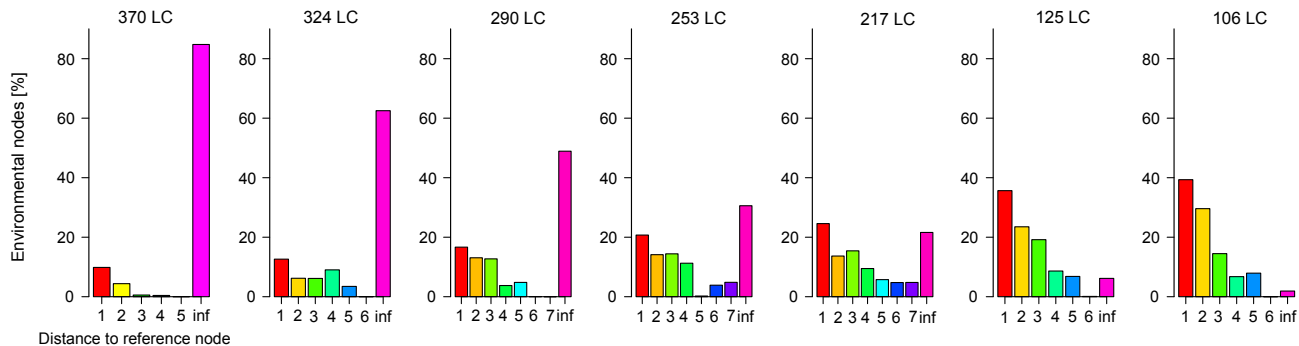

## DNA

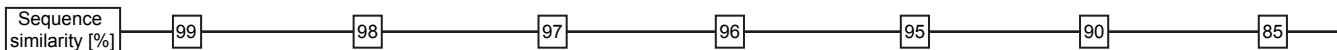

## cDNA

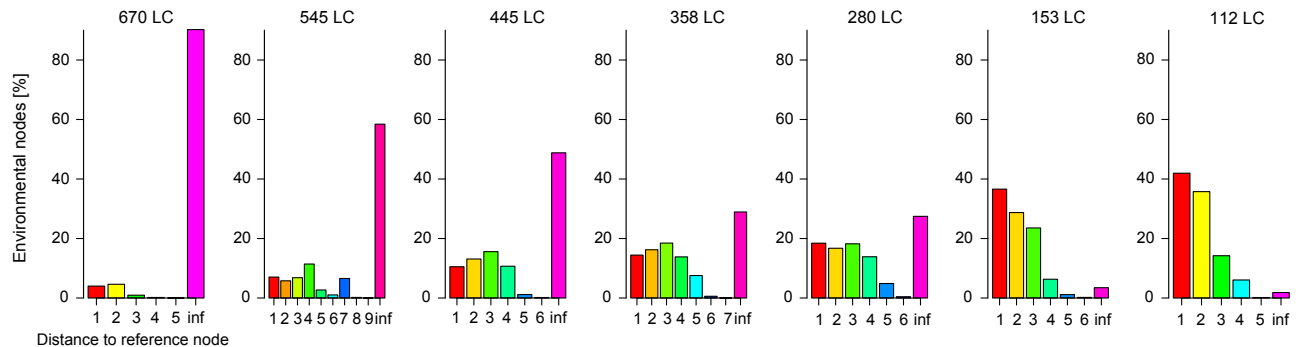

Supplement: Additional file 4: Figure S3. — Network path analyses of LCs. For each environmental node at all sequence similarity thresholds, the shortest path to a reference node (node of a cultured ciliate sequence) has been calculated. Shortest path analyses of LCs revealed similar patterns as the shortest path analyses of CCs (Additional file 3: Figure S2). [file 12915_2015_125_MOESM4_ESM.pdf]

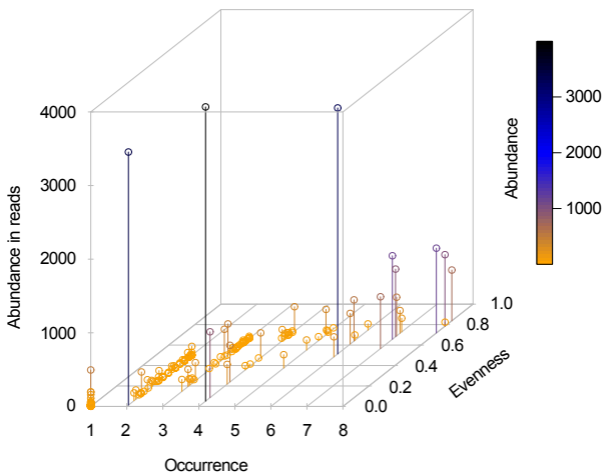

DNA

cDNA

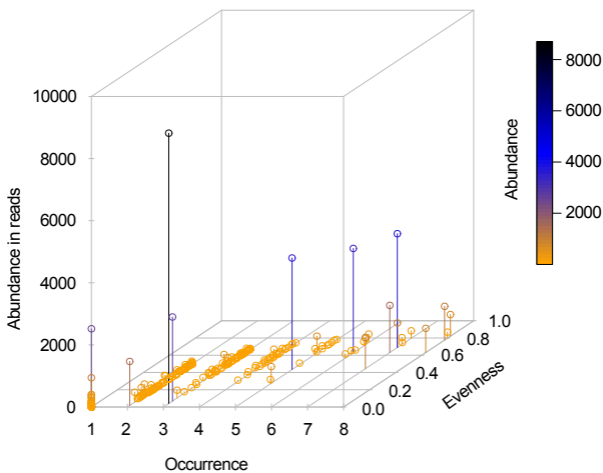

Supplement: Additional file 8: Figure S4. — Three-dimensional plots displaying abundance, occurrence and evenness of sequences in LCs. Plots are based on DNA (above) and cDNA (below) networks at the most exclusive sequence similarity threshold (≥99%). Each dot (370 for DNA; 670 for cDNA) represents one LC. [file 12915_2015_125_MOESM8_ESM.pdf]

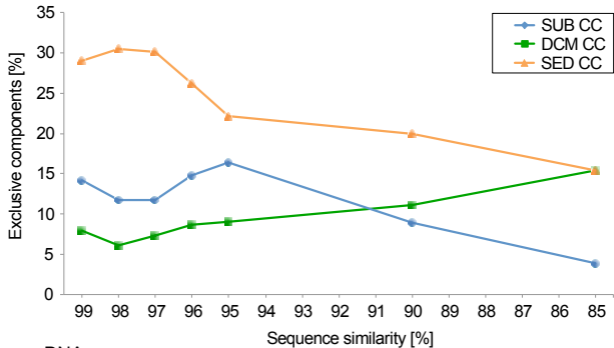

DNA

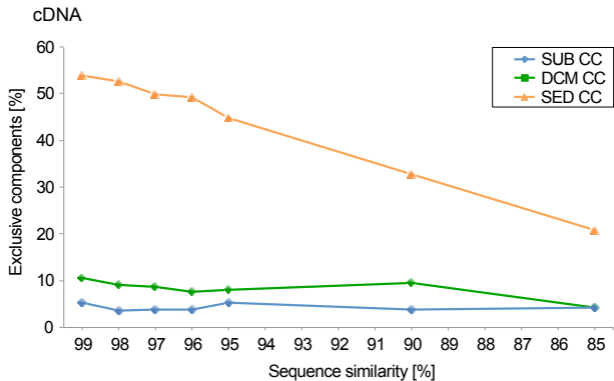

cDNA

Supplement: Additional file 9: Figure S5. — Composition of CC in DNA (above) and cDNA (below) gene similarity networks. Each CC at each similarity threshold was analyzed to highlight the proportion of nodes, which derived from the three investigated habitats in this study (Subsurface, DCM, Sediment). The graphs show the proportion of all CCs, which exclusively consist of nodes of the same habitat. In all networks the highest proportion of exclusive CCs was found for sediment habitats (orange), in some networks providing more than 50% of all CCs. The proportion of DCM (green) and subsurface (blue) CCs never reached more than 15.4% and 16.4%, respectively, of the total number of CCs in the respective network. In the DNA networks an increase of CCs harboring exclusively nodes of DCM habitats could be observed with decreasing sequence similarity. The reason for this is a few exclusive DCM CCs which do not merge with any other CC although the threshold gets more inclusive and fewer CCs in total can be found. Consequently—as the reported values are proportions, not absolute numbers—more CCs can be detected in proportion to the total number of CCs. [file 12915_2015_125_MOESM9_ESM.pdf]
