# Supplementary material for: Testing ecological theories with sequence similarity networks: marine ciliates exhibit similar geographic dispersal patterns as multicellular organisms
Source: BMC Biol. 2015 Feb 24;13:16. doi: 10.1186/s12915-015-0125-5 (PMC4381497; doi:10.1186/s12915-015-0125-5)
Supplement: Additional file 5: Table S2. — Kolmogorov-Smirnov (KS) tests comparing closeness distributions in DNA networks. At all sequence similarity thresholds and for both CCs and LCs two independent KS-tests were performed: 1) to test if the closeness of nodes of cultured ciliates (Ccultured) was higher than the closeness of nodes from former environmental studies before 454 sequencing (Cformer Env) and 2) to test if the closeness of Ccultured was higher than the closeness of all environmental nodes (CEnv: nodes from former environmental studies plus BioMarKs nodes). Closeness can be understood as a measurement of centrality in a network (that is, the higher the closeness, the more central the nodes will be located). For all but one case it could be confirmed that Ccultured was significantly higher than Cformer Env (P <0.05 or P <0.01). Compared to CEnv, Ccultured was always significantly higher. [file 12915_2015_125_MOESM5_ESM.docx]

|  | Connected Components (CC) | | Louvain Communities (LC) | |
| --- | --- | --- | --- | --- |
| Sequence  similarity [%] | C_cultured_ > C_former Env_ | C_cultured_ > C_Env_ | C_cultured_ > C_former Env_ | C_cultured_ > C_Env_ |
| 99 | ** | ** | * | ** |
| 98 | ** | ** | ** | ** |
| 97 | ** | ** | ** | ** |
| 96 | ** | ** | ** | ** |
| 95 | ** | ** | ** | ** |
| 90 | n.s. | ** | ** | ** |
| 85 | ** | ** | ** | ** |

[*] means p<0.05, [**] means p<0.01, [n.s.] means no significance, [C] means Closeness
