# Supplementary material for: Testing ecological theories with sequence similarity networks: marine ciliates exhibit similar geographic dispersal patterns as multicellular organisms
Source: BMC Biol. 2015 Feb 24;13:16. doi: 10.1186/s12915-015-0125-5 (PMC4381497; doi:10.1186/s12915-015-0125-5)
Supplement: Additional file 6: Table S3. — KS-tests comparing closeness distributions in cDNA networks. Results and patterns of the KS-tests on closeness in DNA networks (Additional file 5: Table S2) are confirmed. [file 12915_2015_125_MOESM6_ESM.docx]

|  | Connected Components (CC) | | Louvain Communities (LC) | |
| --- | --- | --- | --- | --- |
| Sequence  similarity [%] | C_cultured_ > C_former Env_ | C_cultured_ > C_Env_ | C_cultured_ > C_former Env_ | C_cultured_ > C_Env_ |
| 99 | * | ** | * | ** |
| 98 | ** | ** | ** | ** |
| 97 | * | ** | ** | ** |
| 96 | * | ** | ** | ** |
| 95 | ** | ** | ** | ** |
| 90 | n.s. | ** | ** | ** |
| 85 | ** | ** | ** | ** |

[*] means p<0.05, [**] means p<0.01, [n.s.] means no significance, [C] means Closeness
