# Supplementary material for: Testing ecological theories with sequence similarity networks: marine ciliates exhibit similar geographic dispersal patterns as multicellular organisms
Source: BMC Biol. 2015 Feb 24;13:16. doi: 10.1186/s12915-015-0125-5 (PMC4381497; doi:10.1186/s12915-015-0125-5)
Supplement: Additional file 7: Table S4. — KS-test comparing the distribution of assortativity in gene similarity networks. Assortativity describes the tendency of nodes to be connected to nodes of the same label. Two independent groups of labels (three habitats, eight locations) were tested for DNA and cDNA networks at each sequence similarity threshold. Using a one-sided KS-test we analyzed if the distribution of assortativity values of each respective label was significantly greater (P <0.05) than expected by chance. Thus the nodes of the respective label were more likely to connect with each other than to connect to nodes of another label. [file 12915_2015_125_MOESM7_ESM.docx]

|  | DNA | | cDNA | |
| --- | --- | --- | --- | --- |
|  | *Investigated*  *habitats (n=3)* | *Investigated*  *locations (n=8)* | *Investigated*  *habitats (n=3)* | *Investigated*  *locations (n=8)* |
| Sequence  similarity [%] | ...of which were significantly assortative | ...of which were significantly assortative | ...of which were significantly assortative | ...of which were significantly assortative |
| 99 | 3 | 5 | 3 | 8 |
| 98 | 3 | 5 | 3 | 7 |
| 97 | 2 | 5 | 3 | 8 |
| 96 | 3 | 5 | 3 | 8 |
| 95 | 3 | 4 | 3 | 6 |
| 90 | 2 | 6 | 2 | 2 |
| 85 | 2 | 6 | 2 | 2 |
